# Interlayer Cation Exchange Stabilizes Polar Perovskite Surfaces

**DOI:** 10.1002/adma.201401858

**Published:** 2014-09-05

**Authors:** Daniel E E Deacon-Smith, David O Scanlon, C Richard A Catlow, Alexey A Sokol, Scott M Woodley

**Affiliations:** University College London, Kathleen Lonsdale Materials Chemistry20 Gordon Street, London, WC1H 0AJ, UK; Diamond Light Source Ltd., Diamond House, Harwell Science and Innovation CampusDidcot, Oxfordshire, OX11 0DE, UK

**Keywords:** potassium tantalate, surface reconstruction, polar surfaces, structure predictions, global optimization

Perovskite oxides are attracting a widespread and growing interest owing to the unique electronic properties they exhibit including colossal magnetoresistance, high-temperature superconductivity, multiferroicity, transparency, and large temperature-dependent dielectric constants, all of which are either greatly affected or even induced by heterostructure interfaces and surfaces.^1^–^6^ These properties give the materials great potential in the emerging field of oxide electronics, allowing the creation of tunable dielectric capacitors and nanosized transistors.^7^,^8^ With the recent discovery of the two-dimensional electron gas (2DEG) on the cleaved (001) surfaces of KTaO_3_ and SrTiO_3_, perovskite surfaces are now the focus of much increased attention.^9^–^12^ Using angle-resolved photoemission spectroscopy (ARPES), King et al.^9^ observed the 2DEG upon cleaving the (001) surface in vacuum from both doped and undoped single-crystal KTaO_3_ samples. Two other studies, however, showed the KTaO_3_ surface to be metallic only after Ar^+^ irradiation in vacuum.^13^,^14^ Harashima et al.^13^ used a five-probe Hall method to measure the Hall effect and conductivity of their (001) single-crystal cut surface samples (preparation conditions unspecified), while Kubacki et al.^14^ used X-ray photoelectron spectroscopy on single-crystal (001) surfaces obtained by epi-polishing. Kubacki et al. went on to show that the metallic state can be removed by annealing in oxygen at 300 °C, although a modified band structure is still present. These differing reports suggest that the preparation procedures used in creating the surface, which determines its structure and composition, are vital in the emergence of these novel properties.

At present, however, the precise experimental structure of (001) KTaO_3_ surface is still uncertain. Helium-atom scattering experiments have demonstrated that initially, after cleaving, (2 × 1) metastable reconstructions can appear, eventually decaying to a (1 × 1) surface structure if heated above 330 K.^15^,^16^ Secondary-ion mass spectrometry on KTaO_3_ surfaces heated to 800 °C and 900 °C in oxidizing conditions reveals a pronounced cation segregation close to the surface, resulting in a non-perovskite phase.^17^ KO is shown to be dominant at the top of the surface, while TaO_2_ is more abundant in the deeper subsurface layers. Similar cation segregation has also been observed at the surface of many other perovskites, notably KNbO_3_, SrTiO_3_, PbTiO_3_, BaTiO_3_.^18^–^21^ Previous computational studies using density functional theory (DFT) considered only a limited number of surface arrangements, reporting a KO terminated, (2 × 1)-terraced surface to be the most stable for stoichiometric KTaO_3_.^22^ For such a complex system, however, it is essential to explore a full range of structural possibilities systematically to establish a robust and reliable model.

**Figure 1:**
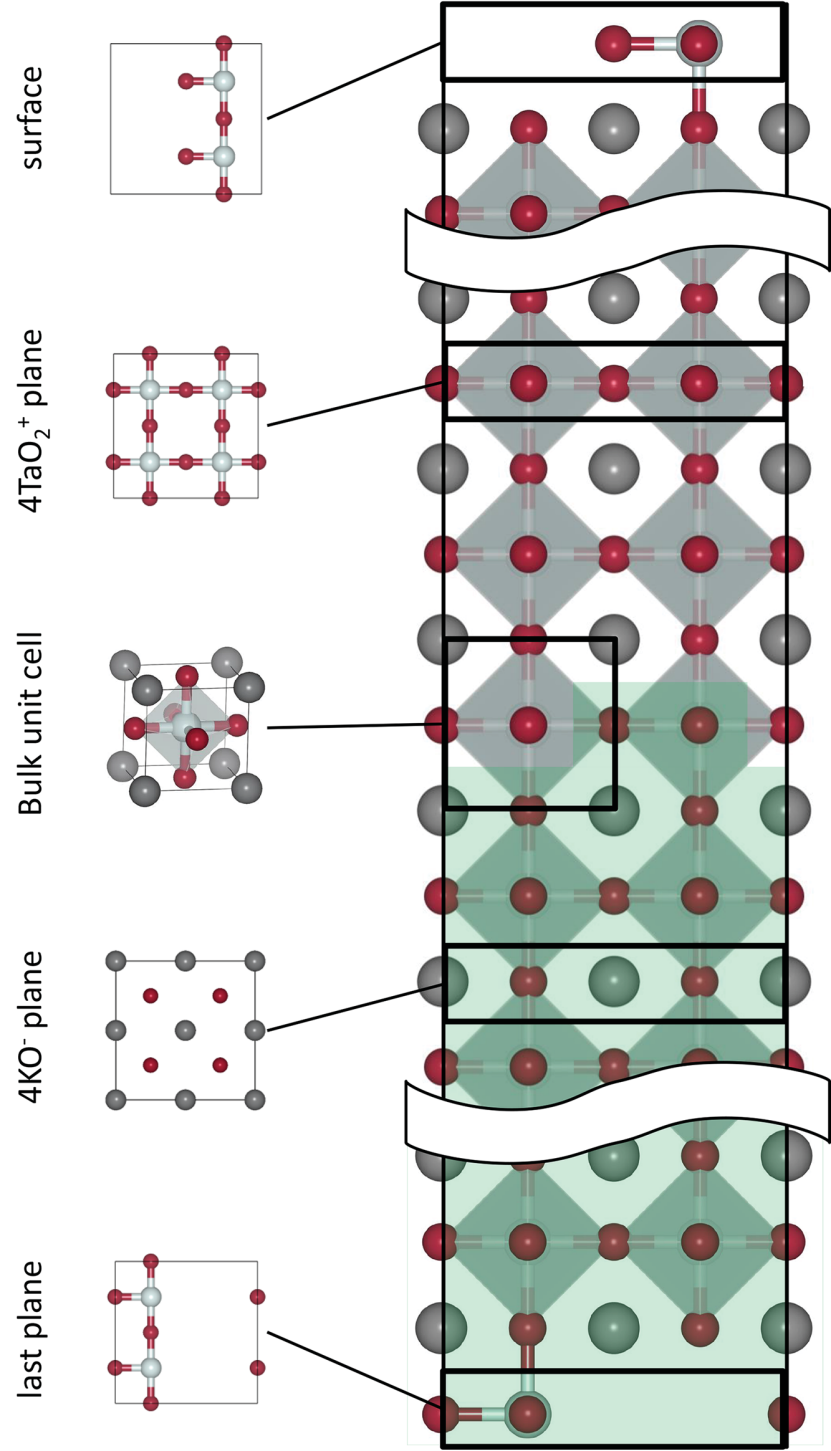
Slab model. An example two-region slab model of a (001) KTaO_3_ surface, with a zero dipole, showing the alternating charged layers and the structure of the bulk unit cell. Panelson the left highlight the different charged layers and the structure of the bulk unit cell. Atoms in the green zone (lower region) are fixed during relaxations.

Below its melting point, KTaO_3_ is a cubic perovskite, which consists of TaO_6_corner-sharing octahedra and K occupying the large dodecahedral sites.^23^ The formal charges for K, Ta, and O are +1, +5, and −2, respectively, and thus along the (001) direction there are alternating charged planes of KO^−^ and TaO_2_^+^ (**Figure** **1**), yielding stoichiometric slabs with “Tasker type 3” polar surfaces, which are unstable and must undergo reconstruction.^24^

Here, we report a new surface stabilization mechanism, supported by a range of reconstructions of the 2 × 2 stoichiometric KTaO_3_ (001) surface, which are more stable than any previously predicted. Our approach employs a combination of atomistic level simulations using interatomic potentials with the lowest energy configurations being refined by DFT.^25^–^28^ By performing a global structure optimization of the KTaO_3_ surface, we show that a reconstruction of the TaO_2_ terminated surface, results in a set of surfaces that are more stable than any generated reconstructions of the KO terminated surface, including those previously considered.^22^,^29^–^40^ For both metal oxide terminations the topmost surface layer, however, consists of KO units, consistent with experimental reports. As our simulations start with the ideal TaO_2_ termination, we find that K ions migrate upwards from the subsurface into the surface layer, while TaO units descend from the surface into the sub-surface layer. The mechanism is driven by the migration of the highly charged small Ta cation from the surface to the bulk where it gains a higher coordination. This mechanism is, we suggest, general in surface reconstructions, leading to the most stable surface reconstructions of materials that are composed of cations whose charge differs greatly.^41^ Moreover, similar phenomena have been previously noted in different materials, for example, see ref.^42^

For the atomistic simulations, we employ specifically designed interatomic potentials (IP) with parameters fitted using the General Utility Lattice Program (GULP) (see Supporting Information).^43^,^44^ Using IP models allowed us to screen the vast number of possible surface arrangements created by our in-house global optimization code, the Knowledge Led Master Controller (KLMC), and identify the lowest energy, stable configurations (see Supporting Information).^45^

**Figure 2:**
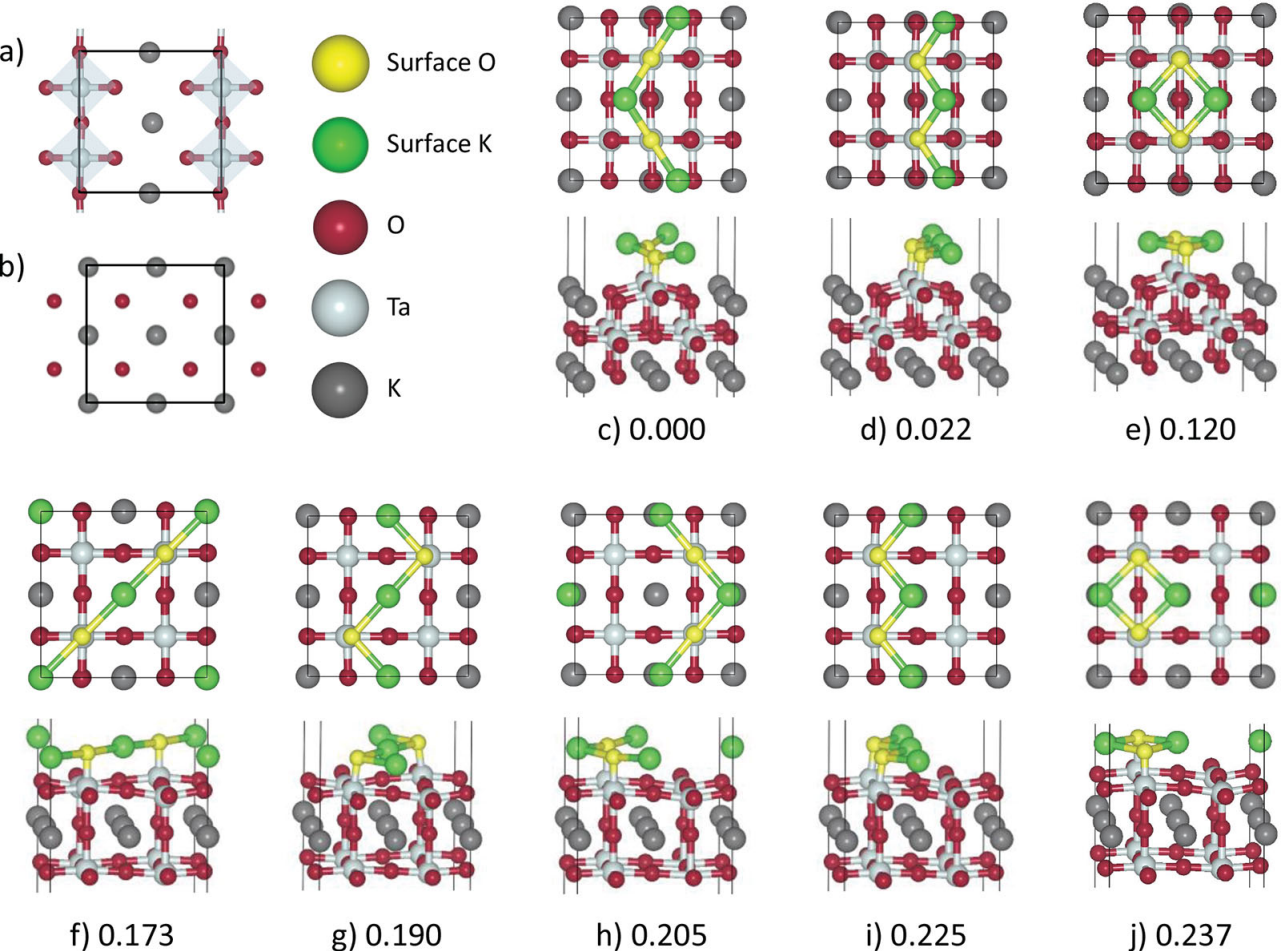
DFT relaxed KTaO_3_ surface structures. a) The mixed subsurface layer obtained by the reconstruction of the TaO_2_ terminated surface; b) pure subsurface layer of the ideal TaO_2_ termination; c–j) eight candidate surface models after DFT relaxation (the top and side views). TaO_2_ terminated surfaces shown in (c–e) where the topmost surface layer (TaO_2_) has mixed with the sublayer (KO), resulting in a new external layer of KO and a mixed sublayer highlighted in (a), consisting of rows of both TaO_2_ and KO. The atomic arrangement of the sublayer and the position of Oionsin the external layer (each directly above a Ta ion in the sublayer)are the same in all three configurations (c–e). These surface models differ only in the arrangement of the K ions around the O ions in the external layer. (f–j) are reconstructions of the KO terminated surface. In these models, the sublayer is an unchanged bulk TaO_2_ layer. The O ions in the external layer are again positioned directly above the Ta ions, however, there are now four possible sites for each pair of O ions to occupy, which results in a more diverse range of surface structures. In total, there are five unique external layers labelled: (c,h) K-cornered –K–O–K–O– zigzag chains; (d,i) KO bulk-like terraces; (e,j) K_2_O_2_ islands; (f) diagonal –K–O–K–O– linear chains; and (g) O-cornered –K–O–K–O–zigzags. Surface energies are relative to the ground state energy,which was found to be 0.871 J m^−2^.

**Figure 3:**
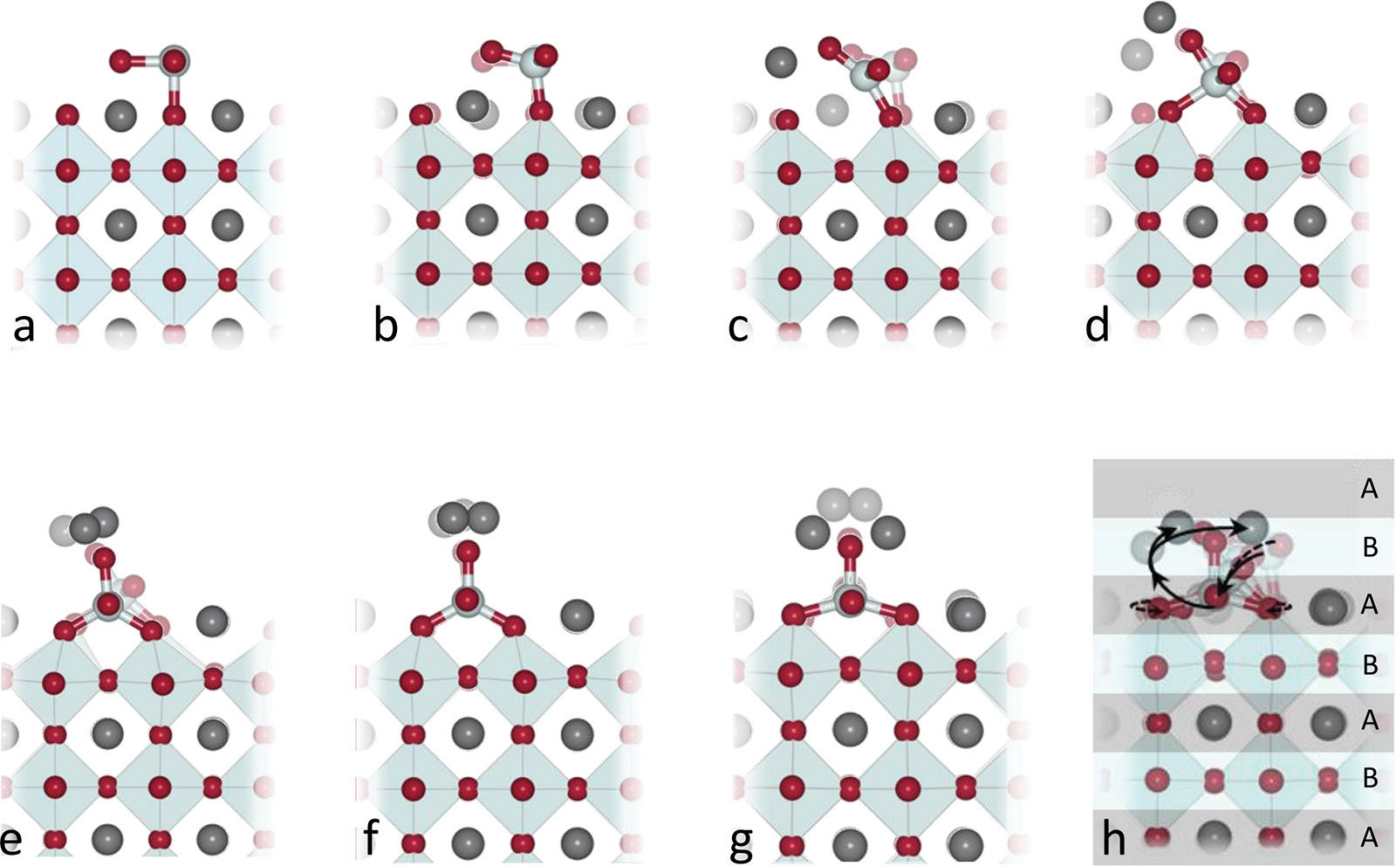
Reconstruction mechanism. Proposed mechanism for the reconstruction of the Ta terminated (001) surface, with K migrating towards the surface, and Ta falling into the sublayer. a) starting bulk terminated configuration. b–g) snap shots with a shadow of the previous frame, with (g) the final DFT relaxed structure. h) the overall DFT transition, where A and B cation layers are shaded differently.

The global optimization revealed that the most stable structures of the TaO_2_ terminated slab have lower surface energies than those of the KO termination (**Figure** **2**). The energetically most favorable reconstructions of the TaO_2_ terminated slab involved a rearrangement of both the upper and lower surface layer. In the support layer, every other chain of K ions is replaced by a TaO chain, so that the 2 × 2 KO bulk-like square lattice layer is transformed into alternating 2 × 1 strips of bulk-like KO and TaO_2_ (**Figure** **3**). The remaining O ions cap the underlying Ta, so forming chains in the upper surface layer, whereas the K ions occupy sites at a maximum distance from cations in the sublayer and with a maximum number of nearest neighbor O ions. In the most stable of these configurations (Figure 2c), K ions arrange themselves to form –K–O–K–O– chains that zigzag with each K ion at a corner. In contrast, the KO terminated slab reconstructions resulted in rearrangements of only the surface layer, as the initial bulk subsurface layer proves to be the most stable configuration.

Our IP search produced two stable configurations of the surface atoms for the TaO_2_-terminated surface, the K cornered zigzag and island surface structures (Figure 2c,e). For the KO-terminated surface there are four stable arrangements: the diagonal, the O-cornered zigzag, the K-cornered zigzag, and the island (Figure 2f–h,j).

The KO bulk-like terraced surface layer (Figure 2d,i) was found to be unstable with IP, and when relaxed adopted the K-cornered zigzag arrangement. We note that when analyzing a 2 × 1 surface, the KO bulk-like terraced surface may appear as stable, as the symmetry breaking of the K cornered surface cannot take place on a 2 × 1 surface, which is indeed applicable to all of our non-bulk-like surfaces. Phonon calculations on the K cornered surface show a low frequency surface mode, with surface K ions vibrating in the direction that would lead to a phase transition between our ground state and the KO bulk-like surface.

Next, the eight surface structures (six of which were found by global optimization on the IP landscape, the other two were obtained by cleaving, which resulted in terraced bulk-like terminated surfaces) have been relaxed using DFT as implemented in the Vienna ab initio Simulation Package (VASP); details are given in the Supporting Information. The DFT calculations support the results of our IP calculations in that the surfaces with the mixed sublayer – initially TaO_2_ terminated – are lower in energy than surfaces with the TaO_2_ sublayer. Although the KO bulk-like terrace surface is now metastable (i.e., a local minimum in energy), the K-cornered zigzag surface, to which the terraced structure relaxes using the IP methods, proves to be energetically more favorable, constituting the ground state.

A feature present in all of these surfaces is that the surface O ions are always directly above the tantalum ions in the sublayer. Previous work on the structure prediction of ionic clusters have shown that, given the opportunity, higher charged cations maximize their coordination before the lower charged cations.^46^ This behavior leads to the higher charged cations located nearer the center of clusters, whereas the lower charge cations are forced to take up positions close to the surface. By analogy, it is then unsurprising that the TaO_2_-bulk terminated surface reconstructed in a way such that the tantalum descends into the bulk to become fully coordinated, and that the stable surface arrangements all have oxygen ions above tantalum instead of potassium. Moreover, the diagonal and O-cornered zigzag surface structures are not stable on the mixed sublayer, as these surfaces would be terminated by under-coordinated Ta ions, despite there being sufficient surface O ions to give a full Ta coordination. For the KO bulk terminated surface, we have found five metastable configurations (Figure 2f–j). As there are now four Ta ions in the layer below, the O^2−^ions are no longer constrained to the same row, which allows the formation of the diagonal and O-cornered zigzag structures. The difference in stability of these five configurations can be attributed to the distances and shielding between the like ions, with the O ions separation being somewhat more significant due to their higher charge. However, the effect of completing the Ta coordination of six dwarfs that of the topmost surface layer arrangement, as all of the mixed sublayer surface reconstructions are lower in energy. Thus, we suggest that the driving mechanism for stabilization of the K rich surface is the fulfillment of the Ta coordination due to the large charge disparity between Ta and K.

Turning our attention to the more general area of (I–V) perovskites, we highlight a number of experimental reports where (001) or (110) surfaces have been found rich in group 1 elements.^18^,^20^ A computational simulation of the KNbO_3_ (110) O terminated surface shows the segregation of the K and Nb in the KNbO subsurface layer, with K migrating towards the surface.^47^–^49^

Furthermore, calculations performed on the (110) O-terminated surfaces of BaTiO_3_ and LaMnO_3_, have reported a slight segregation between Ba and Ti in the BaTiO sublayer of BaTiO_3_, Ba moving towards the surface, but almost none in the LaMnO sublayer of LaMnO_3_, giving support to the hypothesis that the charge disparity plays a crucial role in the segregation of cations near the surface.^29^–^31^,^50^

Although the generic tendency of smaller and higher charged cations to move into the bulk, away from the surface, and their drive for an increase in coordination are well established, the realization of their potential for cation exchange with more labile species – such as light alkali ions – in the sub-surface regions is identified here as the primary mechanism in the stabilization of these materials.^41^,^51^ We note that a popular method to deduce ordering in ionic systems based on Madelung potentials/energies fails, highlighting the importance of short-range interactions and polarization for KTaO_3_.

**Figure 4:**
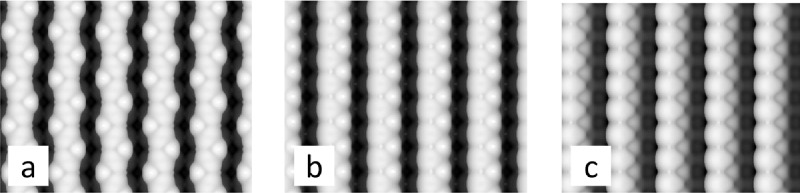
STM images. Simulated STM images (a,b,c) of surfaces in Figure 2c,d,i. The brightness correlates to the distance from the tip. The images are based on charge density isosurfaces obtained by integration over the electron energy bands close to the top of the valence band.^52^

To facilitate future experimental analysis, we have produced simulated scanning tunneling microscopy (STM) images (**Figure** **4**) of three DFT relaxed surfaces: the ground state, and the KO bulk-like surface arrangements on both of our stable surface sublayers. There are presently no high-resolution experimental STM results of the KTaO_3_ (001). As a model for the STM images we show a surface of constant charge density of the electronic states close to the Fermi level, which are probed in experiment. The color indicates the distance from the tip. The images highlight the difficulty in trying to distinguish between the two KO bulk-like terminated surfaces (Figure 4b,c) by STM alone despite the differing sublayers, but highlight the difference in the charge distribution between our ground state (Figure 4a) and the other surface arrangements.

In conclusion, using global optimization techniques, we have uncovered a range of surface arrangements for the stoichiometric (001) KTaO_3_ surface lower in energy than those previously modelled. This includes a new ground state with a surface energy of 0.871 J m^−2^, which is 0.225 J m^−2^ lower in energy than the currently assumed ground state. We propose that the mechanism for the stabilization of KTaO_3_ is driven by the preferential fulfillment of the Ta coordination, due to the large charge disparity between Ta and K. This factor causes the Ta to descend into the bulk for TaO_2_ terminated surfaces, while K migrates to the surface. Having found the most stable reconstruction for the stoichiometric (001) KTaO_3_ surface, future studies based upon this model will address the nature of the yet unexplained electronic phenomena including the formation of the 2DEG. It is clear that the stoichiometric terminations we studied would not support a 2DEG, but the 2DEG could be expected to be a result of electron doping at the surface, with the role of oxygen perhaps being crucial and the actual source of doping remaining very much in question.
