# Supplementary material for: Interlayer Cation Exchange Stabilizes Polar Perovskite Surfaces
Source: Adv Mater. 2014 Sep 5;26(42):7252–6. doi: 10.1002/adma.201401858 (PMC4241033; doi:10.1002/adma.201401858)
Supplement: Supplementary file 1 — Supplementary [file adma0026-7252-SD1.pdf]

Copyright WILEY-VCH Verlag GmbH & Co. KGaA, 69469 Weinheim, Germany, 2014.

ADVANCED MATERIALS

Supporting Information

for *Adv. Mater.*, DOI: 10.1002/adma.201401858

Interlayer Cation Exchange Stabilizes Polar Perovskite Surfaces

*Daniel E. E. Deacon-Smith, David O. Scanlon, C. Richard A. Catlow, Alexey A. Sokol, and Scott M. Woodley**

Supporting Information

Interlayer Cation Exchange Stabilizes Polar Perovskite Surfaces

Daniel E. E. Deacon-Smith, David O. Scanlon, C. Richard A. Catlow, Alexey A. Sokol, and Scott M. Woodley**

Interatomic Potentials – Interatomic potentials for $KTaO_3$ (Table 1) were derived by performing a relaxed fit using the General Utility Lattice Program (GULP). The Buckingham and Lennard-Jones potentials were used to model the short range interaction, and the Coulomb potential between the ions modelled the long range interactions, giving:[1]

$$V_{ij} = A_{ij} e^{-\frac{\rho}{r_{ij}}} + \frac{B}{r_{ij}^{12}} - \frac{C}{r_{ij}^{6}} + \frac{q_i q_j}{r_{ij}} \qquad (1)$$

A, ρ, B, and C were parameters we fit, r_{ij} the distance between ions i and j, and q the charge of each ion. We used the polarisable shell model in GULP to incorporate the polarisation of the ions.[2] The shell model treats each ion as two point charges that are screened from each other, but coupled by a spring. The two point charges split the ion up into a core, the nucleus and the inner electrons, and the valence electron shell. The short range potentials are only allowed to interact with the shell of each ion. The energies associated with spring constants k_2 and k_4 are $k_2 x^2/2!$ and $k_4 x^4/4!$ respectively.

Table 1. Potential parameters for $KTaO_3$. The Lennard-Jones potentials were only used for the rigid ion runs. The charge of the ions in the rigid ion runs for K, Ta, and O were +1, +5, and -2 respectively. A cut-off of 15 Å was used for all potentials.

**Ion**	**Shell charge (e)**	**k_2 (eVÅ^2)**	**k_4 (eV^{2}Å^4)**
K^+	-4.723061	356.91688	20000
Ta^{5+}	-3.700216	68867.76829	10000
O^{2-}	-2.891878	33.45182	33000
	**Buckingham potentials**		
**Interaction**	**A (eV)**	**ρ (Å)**	**C (eV Å^6)**
K – O	1222.43483	0.346334	39.48
Ta – O	1410.97032	0.369327	10.00
O – O [a]	22700.00000	0.149000	113.26
	**Lennard Jones potentials**		
**Interaction**	**B (eVÅ^{12})**		
K – O	10.00		
Ta – O	10.00		

[a] For the O – O interaction the original Buckingham potential of Catlow was modified so that below 2 Å only the repulsive terms were considered, while above 2.6 Å only the attraction was considered, a polynomial fit was used to smooth the potential between the two cut-offs.[3]

We fit the parameters in equation (1) to the room temperature lattice, elastic, and dielectric constants (Table 2), as well as 31 phonon frequencies.[41] Application of the developed interatomic potentials shows excellent agreement of the calculated and experimental lattice parameter, with a difference of less than 0.02%. The dielectric constants calculated using our potentials also show good agreement with experimental data, the static (ε_0) and high frequency (ε_∞) dielectric constants differing by less than 0.01% and 8% respectively. The calculated elastic constants differ by up to 15% from the experiment, a reasonable agreement considering the large number of observables being fit to. The ferroelectric soft mode calculated by our potentials was measured to be 39 cm^{-1} while the ferroelectric mode it was fit to was seen to be 81 cm^{-1}. The ferroelectric mode is observed to fall to 24 cm^{-1} at 4 K. Thus our potentials are softer than expected at the temperature they were fit to (300K), but are harder than what would be expected at low temperature. For our potentials the cubic phase remains stable within a lattice parameter range of 3.95-4.02 Å.

Table 2. Observables. The observables used for fitting the interatomic potentials, lattice parameter (a), elastic constants (C), and dielectric constants (ε).

Observable	Target	Calculated
a (Å)	3.98948 [5]	3.98896
C_{11} (GPa)	396.30 [6]	433.44
C_{12} (GPa)	130.00 [7]	111.69
C_{44} (GPa)	107.10 [6]	111.69
ε_0	243.000 [8]	243.016
ε_∞	4.300 [9]	4.657

Global Optimisation and KLMC – A global structure optimisation was performed using our in-house structure prediction code, the Knowledge Led Master Controller (KLMC), on the $KTaO_3$ surface.[4] We used a one-sided surface model periodic in the a and b directions, and 25 layers (~50 Å) thick in the c direction (Figure 1). To account for the surface reconstruction a 2x2 surface supercell has been employed. The upper thirteen atomic layers are allowed to relax, while the lower 12 were held fixed. We investigated both KO and TaO_2 terminated slabs. The initial slabs were reconstructed to remove the dipole by displacing half of the top atomic layer to beneath the bottom layer. These initial slab models are the same as those previously modelled by Fritsch, where the KO terminated surface was found to be more stable. In the surface structure global optimisation of $KTaO_3$ we employed a Monte Carlo quenching technique. A grid of the bulk lattice positions was created - the ions of the slab all sitting on grid positions before relaxation - and ions were allowed to swap grid positions. The grid extended to one layer above the surface, allowing atoms to take up positions above the surface, and for vacancies to be introduced into the slab. KLMC allowed us to restrict the grid positions involved in the swapping process, thus confining surface reconstructions to the upper layers only. KLMC also enabled us to separate the grid positions into types, making it possible to prevent cations from swapping with anions. Our initial runs limited the

reconstruction (swapping of ions) to the top two layers, with additional runs probing further into the bulk up to 4 layers. For each structure we performed two runs. The first run was a rigid ion run, where the ions were modelled as single point charges. The second run used the output geometry of the first run as its starting structure and used the shell model. After KLMC initialised each new structure, GULP was used to perform a relaxation.

Figure 1. Slab Model. An example slab model of a (001) $KTaO_3$ surface, with a zero dipole, showing the alternating charged layers, and the structure of the bulk unit cell. Also highlighted are the alternating charged layers and the structure of the bulk unit cell.

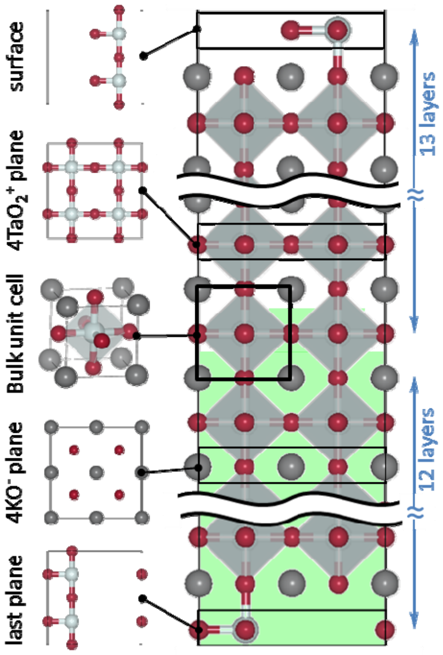

DFT calculations – The VASP calculations were performed using the PBEsol functional, the PAW potentials, and a plane wave cut off of 500 eV.[10-12]. For the bulk calculations to determine the lattice parameter, a k-point mesh of 6 x 6 x 6 was used, while for the surface we used a 3 x 3 x 1 mesh. To calculate the bulk lattice parameter we performed a series of bulk relaxations for differing volumes, and then fitted the energy-volume data using the Birch-Murnaghan equation of states. We obtained a lattice parameter of 3.99215 Å within 0.1% of the experimental result. For the VASP calculations, a two-sided surface model was employed. We used a periodic slab consisting of 17 symmetric layers (~34 Å), totalling 160 atoms, with a vacuum gap of 18 Å. Both the slab thickness and vacuum gap were well converged with respect to surface energy. Surface geometry optimisation calculations were considered converged when the force on each atom was less than 0.01 $eVÅ^{-1}$.

Surface energy - The stability of a material surface is determined by its surface energy:

$$E_{surface} = \frac{E_{slab} - E_{bulk}}{A} \quad (2)$$

where E_{slab} is the relaxed energy of the cleaved system, E_{bulk} is the energy of the bulk containing the same number of atoms as the slab, and A is the surface area of the cell used. When using a double sided slab, the surface energy calculated should be halved as there are two surfaces.

[1] M. T. Dove, *Structure and Dynamics: an Atomic View of Materials*, Oxford Univ., **2003**, 334.

[2] B. G. Dick and A. W. Overhauser, *Physical Review* **1958**, 112, 90-103.

[3] C. R. A. Catlow, *Proceedings of the Royal Society of London. A. Mathematical and Physical Sciences* **1977**, 353, 533-561.

[4] S. M. Woodley, *The Journal of Physical Chemistry C* **2013**, 117, 24003-24014.

[5] P. Vousden, *Acta Crystallographica* **1951**, 4, 373-376.

[6] H. H. Barrett, *Physics Letters A* **1968**, 26, 217-218.

[7] R. Comes, M. Lambert and A. Guinier, *Solid State Communications* **1968**, 6, 715-719.

[8] S. H. Wemple, *Physical Review* **1965**, 137, A1575-A1582.

[9] A. S. Barker and J. J. Hopfield, *Physical Review* **1964**, 135, A1732-A1737.

[10] P. E. Blöchl, *Physical Review B* **1994**, 50, 17953-17979.

[11] G. Kresse and D. Joubert, *Physical Review B* **1999**, 59, 1758-1775.

[12] J. P. Perdew, A. Ruzsinszky, G. I. Csonka, O. A. Vydrov, G. E. Scuseria, L. A. Constantin, X. Zhou and K. Burke, *Physical Review Letters* **2008**, 100, 136406.
